# Reversible heart failure and subsequent bradyarrhythmia during long-term follow-up in a patient with chronic hypocalcemia: a case report

**DOI:** 10.3389/fcvm.2026.1908024

**Published:** 2026-10-21

**Authors:** Ruining Li, Xiangning Deng, Zhongheng Li, Lijie Sun

**Affiliations:** Department of Cardiology, Peking University Third Hospital, NHC Key Laboratory of Cardiovascular Molecular Biology and Regulatory Peptides, Beijing, China

**Keywords:** atrial fibrillation, bradyarrhythmia, heart failure, hypocalcemic cardiomyopathy, hypoparathyroidism

## Abstract

Chronic hypocalcemia is an uncommon, potentially reversible cause of heart failure. Existing reports have mainly described recovery of ventricular function with calcium correction, whereas long-term rhythm outcomes after clinical improvement remain poorly characterized. We report the case of a 74-year-old man with a history of hypocalcemia for more than 40 years and coronary artery disease for 19 years, who was admitted for acute decompensated heart failure. He was ultimately diagnosed with hypocalcemic cardiomyopathy. Following calcium and calcitriol supplementation together with heart failure therapy, his dyspnea resolved, the corrected QT interval shortened, and echocardiography demonstrated improvement in left ventricular ejection fraction. However, 11 months after discharge, he developed persistent atrial fibrillation with a slow ventricular response and intermittent third-degree atrioventricular block, subsequently underwent permanent pacemaker implantation after clinical evaluation. This case highlights the importance of long-term rhythm surveillance after recovery from hypocalcemic cardiomyopathy, while recognizing that late arrhythmic events may be influenced by multiple contributing factors.

## Introduction

Acute decompensated heart failure is often attributed to established cardiovascular disease, but reversible metabolic causes require consideration, especially when ischemia or structural heart disease does not fully explain the presentation. Among these causes, disturbances in calcium homeostasis are uncommon but clinically important because timely recognition can lead to substantial improvement in cardiac function.

Calcium homeostasis is essential for myocardial excitation–contraction coupling. Severe or prolonged hypocalcemia may result in QT interval prolongation, impaired myocardial contractility, and heart failure with reduced ejection fraction. Hypocalcemic cardiomyopathy is often reversible, with reported improvement in symptoms, QTc interval, and left ventricular systolic function after calcium correction and heart failure therapy.

However, biochemical stabilization and cardiac function may not necessarily indicate complete cardiac recovery. Long-term arrhythmic outcomes are less well described. As illustrated by this case, hypocalcemia may exert broader cardiac effects beyond contractile impairment, highlighting the need for continuous rhythm monitoring even after systolic recovery.

## Case description

A 74-year-old male presented with 1 month of progressive exertional dyspnea, oliguria and edema, which worsened to orthopnea one day before admission. He had hypocalcemia for more than 40 years and a 19-year history of coronary artery disease, for which he had undergone percutaneous coronary intervention with stent implantation. He was maintained on secondary prevention therapy including aspirin, clopidogrel, and statin, with clopidogrel discontinued one year after the procedure.

Physical examination revealed evidence of volume overload. Jugular venous distension was noted. Crackles were heard at both lung bases. Heart sounds were muffled, with a regular rhythm and a heart rate of 105 beats per minute. No pathological murmurs were heard. Mild pitting edema was present in both lower extremities. Neurological examination showed normal muscle strength and tone, without myoclonus. Chvostek's and Trousseau's signs were negative.

Laboratory testing revealed an NT-proBNP level of 11,900 pg/mL, cardiac troponin I (cTnI) and creatine kinase-MB (CK–MB) were within reference ranges. The electrocardiogram (Figure 1A) showed sinus tachycardia, complete left bundle branch block (LBBB), and QTc prolongation (505 ms). QTc was automatically calculated using the Bazett correction, with a QRS duration of 136 ms. Echocardiography demonstrated diffuse left ventricular hypokinesis, a left ventricular end-diastolic diameter of 57 mm, and a left ventricular ejection fraction (LVEF) of 33%. Chest x-ray revealed pulmonary edema with small pleural effusions. These findings were consistent with acute decompensation of chronic heart failure, and the patient was admitted to the cardiac intensive care unit.

**Figure 1:**
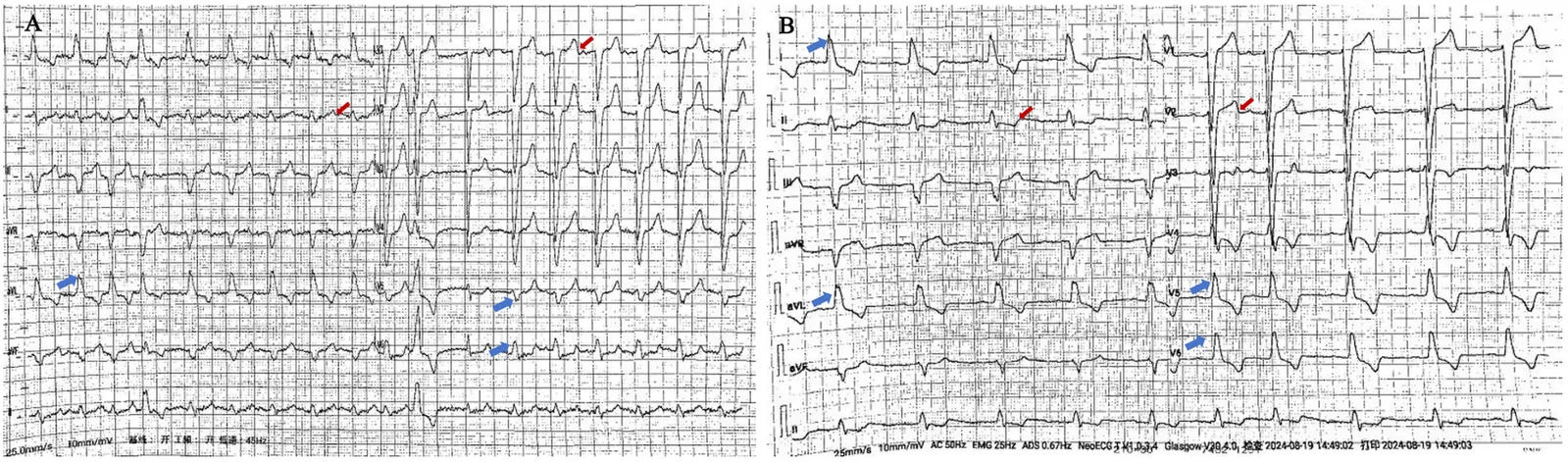
**(A)** the admission ECG revealed QTc prolongation (red arrow) and a left bundle branch block (blue arrow). **(B)** Following the correction of hypocalcemia, the QTc interval essentially shortened prior to discharge (red arrow).

Despite well-controlled cardiovascular risk factors, ischemic cardiomyopathy remained a concern. Coronary angiography was therefore repeated following clinical stabilization, showing no progression of prior lesions and no new significant stenosis. Cardiac magnetic resonance imaging showed mid-wall delayed enhancement in the basal-to-mid interventricular septum, a pattern not typical of ischemic cardiomyopathy. Representative cardiac magnetic resonance images and cine imaging are provided in Supplementary Figure S2 and Supplementary Video S1. These findings made myocardial ischemia unlikely to be the primary driver of the current heart failure decompensation.

Initial laboratory testing demonstrated profound electrolyte disturbances, including severe hypocalcemia (1.04 mmol/L; reference 2.2–2.7 mmol/L), hypomagnesemia (0.63 mmol/L; reference 0.7–1.0 mmol/L), hyperphosphatemia (2.21 mmol/L; reference 0.85–1.51 mmol/L), a parathyroid hormone (PTH) level at the lower limit of normal (15.8 pg/mL; reference 15–65 pg/mL), and mildly reduced 25-hydroxyvitamin D (17.9 ng/mL; reference ≥20 ng/mL). Given the severity of hypocalcemia, the absence of a compensatory increase in PTH suggested hypoparathyroidism. Intracranial calcifications on imaging (Supplementary Figure S1) indicated longstanding calcium–phosphate dysregulation. Primary hypoparathyroidism was considered the underlying cause of hypocalcemia, with repeatedly subnormal PTH levels documented during follow-up.

Calcium and calcitriol supplementation led to normalization of biochemical abnormalities, symptomatic relief, a marked decline in NT-proBNP (Figure 2), QTc shortening (Figure 1B), and improvement in left ventricular systolic function, with LVEF increasing from 33% at admission to 61% at 1-month follow-up (Supplementary Table S1). Collectively, these findings support the diagnosis of hypocalcemic cardiomyopathy.

**Figure 2:**
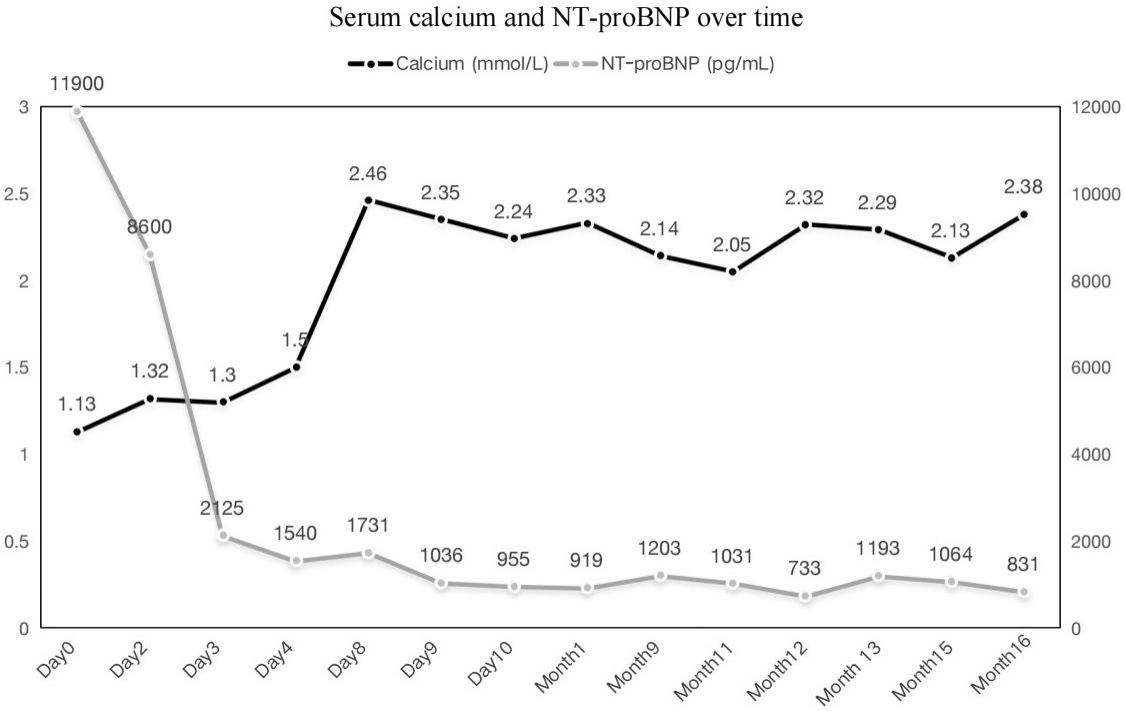
Dynamic trends of serum calcium and NT-proBNP. The horizontal axis indicates time from CCU admission, with the first measurement defined as Day 0 (serum calcium 1.13 mmol/L).

He was followed in the endocrinology clinic for primary hypoparathyroidism with regular monitoring of calcium and phosphate, which remained within the reference range. The calcium carbonate dose was reduced from 1,500 mg to 1,200 mg per day due to joint pain, and calcitriol was gradually decreased from 0.25 µg twice daily to 0.25 and 0.5 µg on alternating days.

Paroxysmal atrial fibrillation was documented approximately 9 months after discharge. Given a CHA₂DS₂-VASc score of 6 and a HAS-BLED score of 2, anticoagulation with rivaroxaban 15 mg once daily was initiated. No pharmacological or electrical cardioversion was performed, and subsequent follow-up demonstrated progression to persistent atrial fibrillation. No beta-blockers, amiodarone, digoxin, or other rate-slowing medications were used before the development of bradycardia. Therefore, medication-induced bradycardia was considered less likely.

Eleven months after discharge, repeated Holter monitoring showed persistent atrial fibrillation with a slow ventricular response (mean ventricular rate 54 bpm; nadir 31 bpm). Notably, daytime ventricular rates below 40 bpm were documented, with the longest R–R interval of 2.25 s. Holter monitoring also documented intermittent third-degree atrioventricular block during atrial fibrillation, with a regular slow ventricular escape rhythm (Figure 3). The patient denied fatigue, dizziness, presyncope, or syncope. Given the documented third-degree atrioventricular block, permanent pacemaker implantation was recommended (1). A dual-chamber permanent pacemaker was implanted, initially programmed in DDD mode. During subsequent follow-up, persistent atrial fibrillation was confirmed, and the pacing mode was adjusted to VVI. Calcium supplementation was adjusted to calcitriol 0.25/0.5 µg on alternating days plus calcium carbonate 1,500 mg per day. Serum calcium, magnesium, potassium, and thyroid function were monitored at the time of bradyarrhythmia evaluation, with no evidence of reversible metabolic abnormalities that could explain the bradyarrhythmia. The timeline of the clinical course is displayed below (Figure 4).

**Figure 3:**
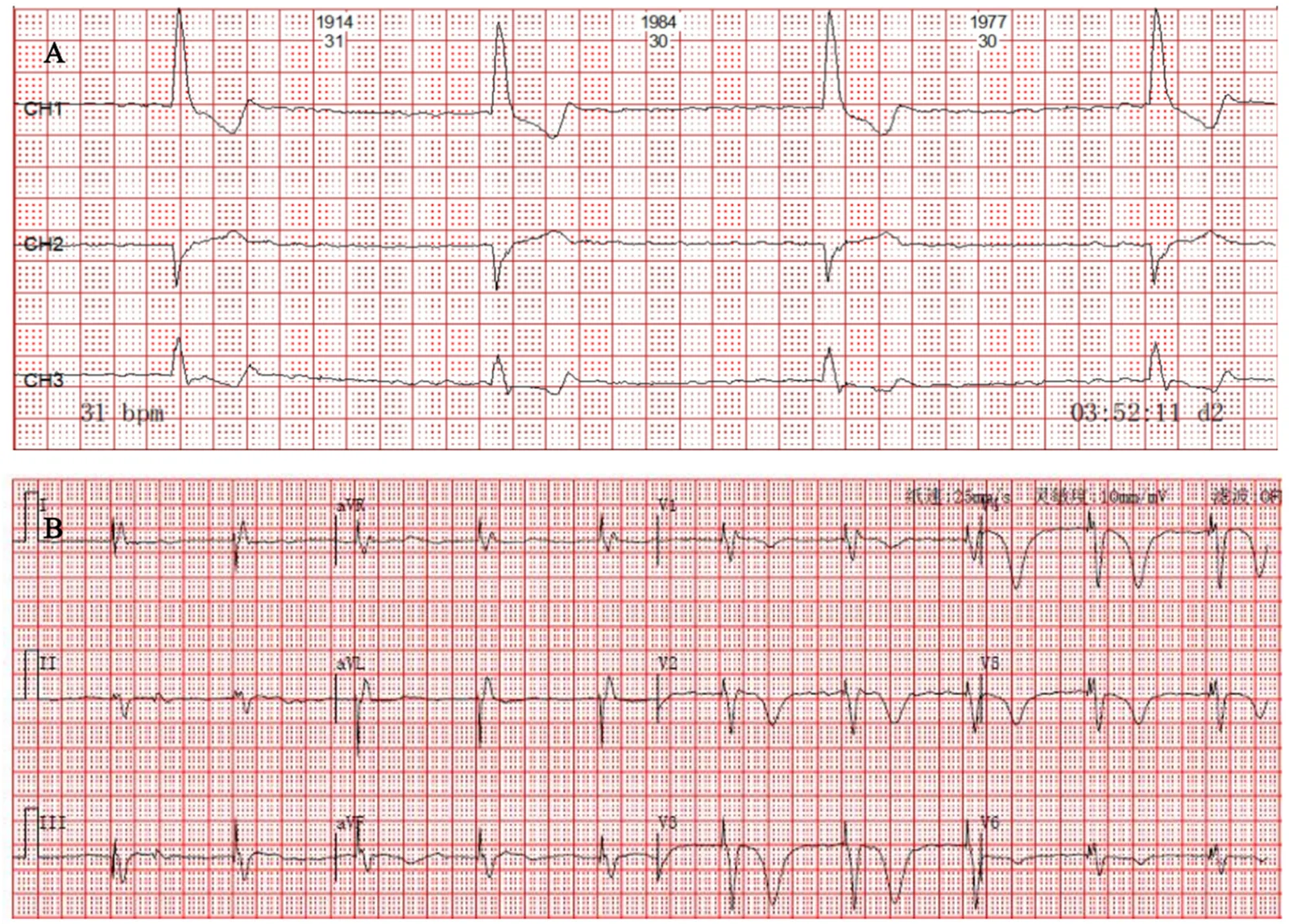
Holter-documented intermittent third-degree atrioventricular block during atrial fibrillation **(A)** and post-pacemaker ECG **(B).**

**Figure 4:**
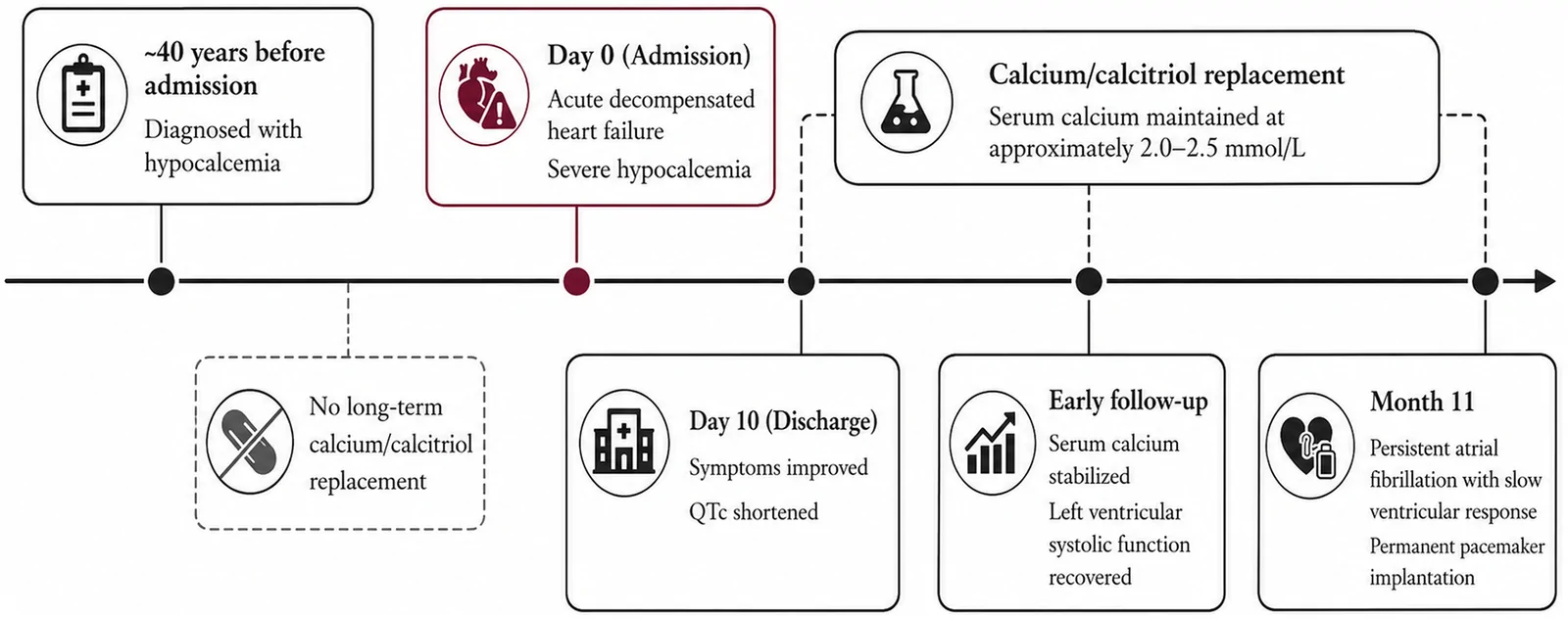
Timeline of the clinical course.

## Discussion

Hypocalcemic cardiomyopathy is an uncommon but reversible cause of heart failure. Our patient had a history of hypocalcemia for more than 40 years before presenting with acute decompensated heart failure. The marked decline in NT-proBNP, shortening of the QTc interval, and increase in LVEF after calcium and calcitriol supplementation were consistent with hypocalcemic cardiomyopathy. However, recovery of left ventricular systolic function should not be taken as evidence of complete myocardial normalization. Xie et al. described a patient with hypocalcemic cardiomyopathy in whom heart failure symptoms subsided and LVEF increased after calcium correction, whereas follow-up cardiac magnetic resonance imaging showed persistent late gadolinium enhancement, suggesting residual myocardial fibrosis (2). These cases suggest that the course of hypocalcemic cardiomyopathy should not be assessed by LVEF alone. Although the admission PTH level was only at the lower limit of normal, it was inappropriately low for the degree of hypocalcemia; coexisting hypomagnesemia may also have blunted PTH secretion (3).

Hypocalcemia may also be associated with electrophysiological abnormalities. Hypocalcemia primarily impaired calcium-dependent inactivation of L-type calcium channels, prolonging action potential duration and manifesting as QT interval prolongation, thereby increasing the risk of malignant ventricular tachyarrhythmias (4). Besides, QTc interpretation in the presence of LBBB requires caution because QRS prolongation may contribute to the measured QT interval. Beyond calcium itself, abnormalities in vitamin D and mineral metabolism may also influence cardiac electrophysiology. Vitamin D signaling in cardiomyocytes has been suggested to regulate calcium-handling proteins and ion channels, providing a potential link between metabolic disturbances and alterations in myocardial repolarization (5). Given magnesium's role in regulating multiple ion transporters, including potassium and calcium channels, hypomagnesemia might adversely affect cardiac conduction and contractile function (6).

Complete LBBB was present on admission and might have contributed to mechanical dyssynchrony, thereby worsening heart failure in the setting of severe hypocalcemia. Only a few cases have described hypocalcemia-associated bundle branch block (7, 8), which normalized after calcium replacement. Atrioventricular block has also been reported in patients with hypocalcemia (9, 10). A focused review of previous reports suggests that rhythm disturbances associated with hypocalcemic cardiomyopathy are most often documented during active hypocalcemia and frequently improve after calcium correction (Supplementary Table S2). A similar pattern has been reported in hypocalcemia-related bradyarrhythmias without definite cardiomyopathy. These cases highlight that hypocalcemia can be accompanied by conduction abnormalities, but do not address whether any irreversible bradyarrhythmia remained, possibly because calcium correction was initiated before persistent conduction-system injury had developed.

Sinoatrial and atrioventricular nodal function depend on calcium currents (11), and severe or prolonged hypocalcemia can suppress sinoatrial automaticity or impair atrioventricular nodal conduction, potentially through disruption of the calcium-clock mechanism in pacemaker cells (12). In our patient, clinically significant bradyarrhythmia occurred months after serum calcium normalization. Currently, it is unclear whether prolonged untreated hypocalcemia contributed to later conduction abnormalities or bradyarrhythmias, including left bundle branch block and slow ventricular rates. Given the patient's advanced age, coronary artery disease, and myocardial fibrosis identified by cardiac magnetic resonance imaging, the later electrophysiological abnormality was possibly multifactorial. Long-standing hypocalcemia may have contributed to increased electrophysiological susceptibility, although a direct causal relationship cannot be established. Metabolic abnormalities have also been implicated in atrial electrical remodeling, with previous studies demonstrating changes in electrocardiographic markers of atrial conduction and electromechanical coupling in patients with metabolic disorders (13).

In conclusion, the cardiac impact of hypocalcemia may extend beyond contractile dysfunction and may be associated with electrophysiological abnormalities during long-term follow-up. Normalization of systolic function should not preclude continued surveillance for arrhythmic complications. Long-term management should include periodic rhythm assessment, while calcium and calcitriol therapy is individualized to maintain biochemical control without increasing the risk of nephrolithiasis or ectopic calcification.

## Data Availability

The original contributions presented in the study are included in the article/Supplementary Material, further inquiries can be directed to the corresponding author.
