# Supplementary material for: Reversible heart failure and subsequent bradyarrhythmia during long-term follow-up in a patient with chronic hypocalcemia: a case report
Source: Front Cardiovasc Med. 2026 Oct 21;13:1908024. doi: 10.3389/fcvm.2026.1908024 (PMC13731214; doi:10.3389/fcvm.2026.1908024)
Supplement: Supplementary file 1 [file Datasheet1.zip › Supplementary Material presentation/Supplementary Table S2.docx]

**Table S2. Reported rhythm and conduction abnormalities associated with hypocalcemia**

**Part A. Hypocalcemic cardiomyopathy with arrhythmia or conduction abnormalities**

| **Reference** | **Case** | **Etiology** | **Cardiac involvement** | **Rhythm or conduction finding** | **Outcome** |
| --- | --- | --- | --- | --- | --- |
| Kudoh et al., 1992^[1]^ | 46-year-old male | Primary hypoparathyroidism; severe long-standing hypocalcemia for at least 20 years. | Heart failure with cardiomyopathy. | Non-sustained ventricular tachycardia, atrial fibrillation, and long QT interval. | Marked improvement in cardiomyopathy after serum calcium correction. |
| Altunbas et al., 2003^[2]^ | Two cases: 55-year-old male and 46-year-old female; arrhythmia documented in the male case. | Post-thyroidectomy hypoparathyroidism; 19 years in male case and 12 years in female case, with intermittent treatment only during symptomatic episodes. | Dilated cardiomyopathy with cardiac failure; normal coronary angiograms; endomyocardial biopsy consistent with cardiomyopathy. | Atrial fibrillation and long QT interval in the 55-year-old male case. | Partial recovery of cardiac function after restoration of normocalcemia. |
| Avsar et al., 2004^[3]^ | 40-year-old female | Post-thyroidectomy hypoparathyroidism; reported duration 3 years. | LVEF around 25% with severe heart failure and mitral regurgitation. | Non-paroxysmal junctional tachycardia and long QT interval. | Clinical and hemodynamic improvement after calcium and vitamin D therapy. |
| Tsironi et al., 2006^[4]^ | 25-year-old male | Beta-thalassemia. | Heart failure associated with hypocalcemia in a thalassemic patient. | Atrial fibrillation and long QT interval. | Outcome not available from the abstract. |
| Tziomalos et al., 2006^[5]^ | 68-year-old male | Primary hypoparathyroidism; duration unknown. | Dilated cardiomyopathy with reduced systolic function. | Atrial fibrillation and long QT interval. | Cardiac dysfunction improved after correction of hypocalcemia. |
| Chavan et al., 2007^[6]^ | 48-year-old male | Vitamin D deficiency; duration unknown. | Cardiomyopathy with ventricular dysfunction in the setting of ventricular tachycardia. | Monomorphic and polymorphic ventricular tachycardia with long QT interval. | Arrhythmia and heart failure resolved with calcium supplementation. |
| Venugopalan et al., 2015^[7]^ | 87-year-old male | Vitamin D deficiency; duration unknown. | Dilated cardiomyopathy with reduced LVEF. | Ventricular tachycardia. | Biochemical abnormalities were corrected with calcium, magnesium, and vitamin D supplementation; symptoms improved and LVEF increased to 55% at 6-month follow-up. |
| Wen and Luo, 2022^[8]^ | 58-year-old female | Postsurgical hypoparathyroidism after thyroidectomy; poor adherence to calcium/vitamin D treatment. | Acute heart failure with LV enlargement and reduced systolic function; LVEF 43% at presentation. | Sinus tachycardia and QTc prolongation at presentation; sinus bradycardia occurred 1 month after discharge and was attributed to ivabradine, resolving after withdrawal. | Calcium and active vitamin D replacement with HF therapy improved symptoms and LVEF. |
| Xie et al., 2023^[9]^ | 52-year-old female | Postsurgical hypoparathyroidism after subtotal thyroidectomy; approximately 11 years since surgery. | Hypocalcemic cardiomyopathy with LVEF 29% and LVEDD 58 mm. | Sinus tachycardia, QTc prolongation, multiple premature atrial complexes with aberrant conduction; Holter showed isolated atrial premature beats, paroxysmal atrial tachycardia, and isolated ventricular premature beats. | Electrolyte correction and HF therapy improved symptoms and LVEF; however, CMR showed persistent LGE. |

**Part B. Hypocalcemia-related bradyarrhythmia or atrioventricular conduction abnormalities**

| **Reference** | **Case** | **Etiology** | **Rhythm or conduction finding** | **Outcome** |
| --- | --- | --- | --- | --- |
| Stefanaki et al., 2005^[10]^ | Preterm infants; pediatric case report and literature review. | Hypocalcemia in preterm infants. | Atrioventricular block associated with hypocalcemia. | AV block improved after calcium correction. |
| Aktas and Akiyama, 2006^[11]^ | 82-year-old male | Hungry bone syndrome related to metastatic prostate cancer, with severe hypocalcemia and vitamin D deficiency. | Wenckebach second-degree AV block, transient third-degree AV block, marked QTc prolongation, and a short episode of torsade de pointes. | Calcium and calcitriol supplementation corrected hypocalcemia; no recurrent torsade de pointes on Holter monitoring; discharged in sinus rhythm without pacemaker implantation. |
| Bitar et al., 2013^[12]^ | 14-year-old female | Osteomalacia-induced severe hypocalcemia; serum calcium 1.12 mmol/L. | Bradycardia, markedly prolonged QTc, and second-degree AV block with 2:1 conduction. | Intravenous calcium gluconate led to disappearance of AV block within 4 hours; QT interval normalized after 7 days. |
| Azak et al., 2017^[13]^ | 2-day-old premature neonate | Hypocalcemia; total calcium 4.8 mg/dL. | 2:1 AV block with prolonged QTc. | Intravenous calcium gluconate was followed by ECG normalization, heart rate recovery, clinical improvement, and LVEF increase to 75%. |
| Santini et al., 2024^[14]^ | 80-year-old female | Iatrogenic hypoparathyroidism after thyroidectomy; recently stopped calcitriol; serum calcium 1.65 mmol/L. | Sinus rhythm around 50/min with episodes of sinus arrest and AV nodal escape rhythm causing symptomatic bradycardia. | Correction of hypocalcemia restored sinus node automaticity; bradycardia and dizziness resolved; calcitriol reintroduced and 24-hour ECG follow-up recommended. |

Abbreviations: AF, atrial fibrillation; AV, atrioventricular; CMR, cardiac magnetic resonance; ECG, electrocardiogram; HC, hypocalcemic cardiomyopathy; HF, heart failure; LGE, late gadolinium enhancement; LVEDD, left ventricular end-diastolic diameter; LVEF, left ventricular ejection fraction; QTc, corrected QT interval; VT, ventricular tachycardia.

**References**

1.	Kudoh C, Tanaka S, Marusaki S, Takahashi N, Miyazaki Y, Yoshioka N, et al. Hypocalcemic cardiomyopathy in a patient with idiopathic hypoparathyroidism. Intern Med. 1992;31(4):561-8. doi: 10.2169/internalmedicine.31.561

2.	Altunbaş H, Balci MK, Yazicioğlu G, Semiz E, Ozbilim G, Karayalçin U. Hypocalcemic cardiomyopathy due to untreated hypoparathyroidism. Horm Res. 2003;59(4):201-4. doi: 10.1159/000069324

3.	Avsar A, Dogan A, Tavli T. A rare cause of reversible dilated cardiomyopathy: hypocalcemia. Echocardiography. 2004;21(7):609-12. doi: 10.1111/j.0742-2822.2004.03149.x

4.	Tsironi M, Korovesis K, Farmakis D, Deftereos S, Aessopos A. Hypocalcemic heart failure in thalassemic patients. Int J Hematol. 2006;83(4):314-7. doi: 10.1532/ijh97.E0532

5.	Tziomalos K, Kakavas N, Kountana E, Harsoulis F, Basayannis E. Reversible dilated hypocalcaemic cardiomyopathy in a patient with primary hypoparathyroidism. Clin Endocrinol (Oxf). 2006;64(6):717-8. doi: 10.1111/j.1365-2265.2006.02538.x

6.	Chavan CB, Sharada K, Rao HB, Narsimhan C. Hypocalcemia as a cause of reversible cardiomyopathy with ventricular tachycardia. Ann Intern Med. 2007;146(7):541-2. doi: 10.7326/0003-4819-146-7-200704030-00020

7.	Venugopalan G, Navinath M, Pradeep B, Sobia N, Chandan Jyoti D, Nitish N, et al. Hypocalcemic Cardiomyopathy Due to Vitamin D Deficiency in a Very Old Man. J Am Geriatr Soc. 2015;63(8):1708-9. doi: 10.1111/jgs.13586

8.	Wen Y, Luo X. Hypocalcemic cardiomyopathy: A case report. Front Cardiovasc Med. 2022;9:999550. doi: 10.3389/fcvm.2022.999550

9.	Xie Y, Yang J, Li K, Liu F, Yi Y, Zhang P. Case report: A case of hypocalcemic cardiomyopathy with non-reversible cardiac fibrosis. Front Cardiovasc Med. 2023;10:1166600. doi: 10.3389/fcvm.2023.1166600

10.	Stefanaki E, Koropuli M, Stefanaki S, Tsilimigaki A. Atrioventricular block in preterm infants caused by hypocalcaemia: a case report and review of the literature. Eur J Obstet Gynecol Reprod Biol. 2005;120(1):115-6. doi: 10.1016/j.ejogrb.2004.09.012

11.	Aktas MK, Akiyama T. Hypocalcemic heart block and torsade de pointes. Folia Cardiol. 2006;13:2.

12.	Bitar ZI, Maadarani O, Alfayed H, Bitar M. Beyond a prolonged QT interval. JRSM Short Rep. 2013;4(11):2042533313494376. doi: 10.1177/2042533313494376

13.	Azak E, Tatar Aksoy H, Ünsal H, Çetin İ. A rare cause of 2:1 atrioventricular block and congestive heart failure in preterm infants: Hypocalcemia. Turk Kardiyol Dern Ars. 2017;45(1):82-4. doi: 10.5543/tkda.2016.82453

14.	Santini M, Schneider L, Bištirlić M, Santini J, Verunica N, Lovrić-Benčić M, et al. Case report: a unique case of sinus arrest and consequent symptomatic bradycardia caused by severe iatrogenic hypocalcemia. Arrhythmology and electrostimulation. 2024.
