# Supplementary material for: Reversible heart failure and subsequent bradyarrhythmia during long-term follow-up in a patient with chronic hypocalcemia: a case report
Source: Front Cardiovasc Med. 2026 Oct 21;13:1908024. doi: 10.3389/fcvm.2026.1908024 (PMC13731214; doi:10.3389/fcvm.2026.1908024)
Supplement: Supplementary file 1 [file Datasheet1.zip › Supplementary Material presentation/Supplementary Material 1 CARE checklist.docx]

**CARE Checklist for Case Report**

**Manuscript title:** Reversible Heart Failure and Late-Onset Bradyarrhythmia in Hypocalcemia: A Case Report

**Article type:** Case Report

| **Topic** | **Location in manuscript** | **Status** |
| --- | --- | --- |
| Title | Title page: “Reversible Heart Failure and Late-Onset Bradyarrhythmia in Hypocalcemia: A Case Report”. | Reported |
| Keywords | Keywords: Heart failure; Hypocalcemic cardiomyopathy; Hypoparathyroidism; Bradyarrhythmia; Atrial fibrillation. | Reported |
| Abstract | Abstract | Reported |
| Introduction | Introduction section, paragraphs 1–3. | Reported |
| Case description | Case description, physical examination, other clinical findings, relevant past interventions | Reported |
| Timeline | Figure 4: Timeline of the clinical course. | Reported |
| Diagnostic assessment | Case presentation: laboratory testing, electrocardiography, echocardiography, chest X-ray, coronary angiography, cardiac magnetic resonance imaging, cranial computed tomography. | Reported |
| Diagnostic assessment | Case presentation: ischemic cardiomyopathy considered and reassessed using coronary angiography and cardiac magnetic resonance imaging; hypoparathyroidism considered based on severe hypocalcemia and inappropriately low PTH. | Reported |
| Diagnostic assessment | Case presentation and Discussion: severe longstanding hypocalcemia, QTc prolongation, left bundle branch block, reduced LVEF, and delayed bradyarrhythmia during follow-up. | Reported |
| Therapeutic intervention | Case presentation: calcium and calcitriol supplementation, heart failure therapy, endocrine follow-up, and permanent pacemaker implantation. | Reported |
| Therapeutic intervention | Case presentation: calcium carbonate and calcitriol dosing and subsequent dose adjustments; permanent pacemaker implantation. | Reported |
| Therapeutic intervention | Case presentation: calcium carbonate dose reduced due to joint pain; calcitriol adjusted during follow-up; therapy adjusted again after pacemaker admission. | Reported |
| Follow-up and outcomes | Case presentation: dyspnea resolved, biochemical abnormalities normalized, NT-proBNP decreased, QTc shortened, LVEF improved, and delayed bradyarrhythmia developed. Patient denied syncope. | Reported |
| Follow-up and outcomes | Case presentation: follow-up echocardiography, Holter monitoring, pacemaker implantation, and serial biochemical monitoring. | Reported |
| Follow-up and outcomes | Case presentation: prior lack of sustained calcium replacement and later regular endocrine follow-up; joint pain prompted calcium dose reduction. | Reported |
| Follow-up and outcomes | Case presentation: persistent atrial fibrillation with slow ventricular response 11 months after discharge, requiring permanent pacemaker implantation. | Reported |
| Discussion | Discussion: novelty emphasized through comparison with previous reports. | Reported |
| Discussion | Discussion: hypocalcemic cardiomyopathy, hypocalcemia-related arrhythmias/conduction abnormalities, magnesium disorders, calcium-channel mechanisms, and focused literature review (Supplementary Table S2). | Reported |
| Discussion | Discussion: relationship between hypocalcemia, QTc prolongation, myocardial recovery, conduction abnormalities, and delayed bradyarrhythmia; underlying conduction system disease acknowledged as not excludable. | Reported |
| Discussion | Discussion, concluding paragraph: systolic recovery should not preclude rhythm surveillance; long-term management should individualize calcium/calcitriol therapy. | Reported |
| Patient perspective | Not reported in the current manuscript. | Not reported |
| Informed consent | Declarations, Consent for publication: written informed consent was obtained from the patient for publication of this case report. | Reported |

Abbreviations used in this checklist: ECG, electrocardiogram; LVEF, left ventricular ejection fraction; NT-proBNP, N-terminal pro-B-type natriuretic peptide; PTH, parathyroid hormone; QTc, corrected QT interval.
