# Supplementary material for: Reversible heart failure and subsequent bradyarrhythmia during long-term follow-up in a patient with chronic hypocalcemia: a case report
Source: Front Cardiovasc Med. 2026 Oct 21;13:1908024. doi: 10.3389/fcvm.2026.1908024 (PMC13731214; doi:10.3389/fcvm.2026.1908024)
Supplement: Supplementary file 1 [file Datasheet1.zip › Supplementary Material presentation/Supplementary Table S1.docx]

Supplementary Table S1. Echocardiographic parameters at baseline and follow-up.

|  | 5Y prior | Day 0 | Day 3 | Day 5 | Month 1 | Month 9 | Month 11 |
| --- | --- | --- | --- | --- | --- | --- | --- |
| LA (mm) | 42 | 44 | 42.5 | 39 | 47 | 45.2 | 44 |
| LVEDD (mm) | 48.6 | 64.6 | 57 | 56 | 54 | 50.5 | 51 |
| IVSD (mm) | 11 | 24.1 | 15 | 16 | 14 | 18.8 | 18 |
| LVEF (%) | 60 | 35 | 33 | 33 | 61 | 60 | 64 |
| RVEDD (mm) | 20.3 | 19 | 21 | 21 | 19 | 20.6 | 18 |
| Right atrial area (cm^2^) | 18 | 26 | 14 | 14 | 16 | 20.6 | 26 |
