# Supplementary material for: Reversible heart failure and subsequent bradyarrhythmia during long-term follow-up in a patient with chronic hypocalcemia: a case report
Source: Front Cardiovasc Med. 2026 Oct 21;13:1908024. doi: 10.3389/fcvm.2026.1908024 (PMC13731214; doi:10.3389/fcvm.2026.1908024)
Supplement: Supplementary file 1 [file Datasheet1.zip › Supplementary Material presentation/Supplementary Figure S2.pdf]

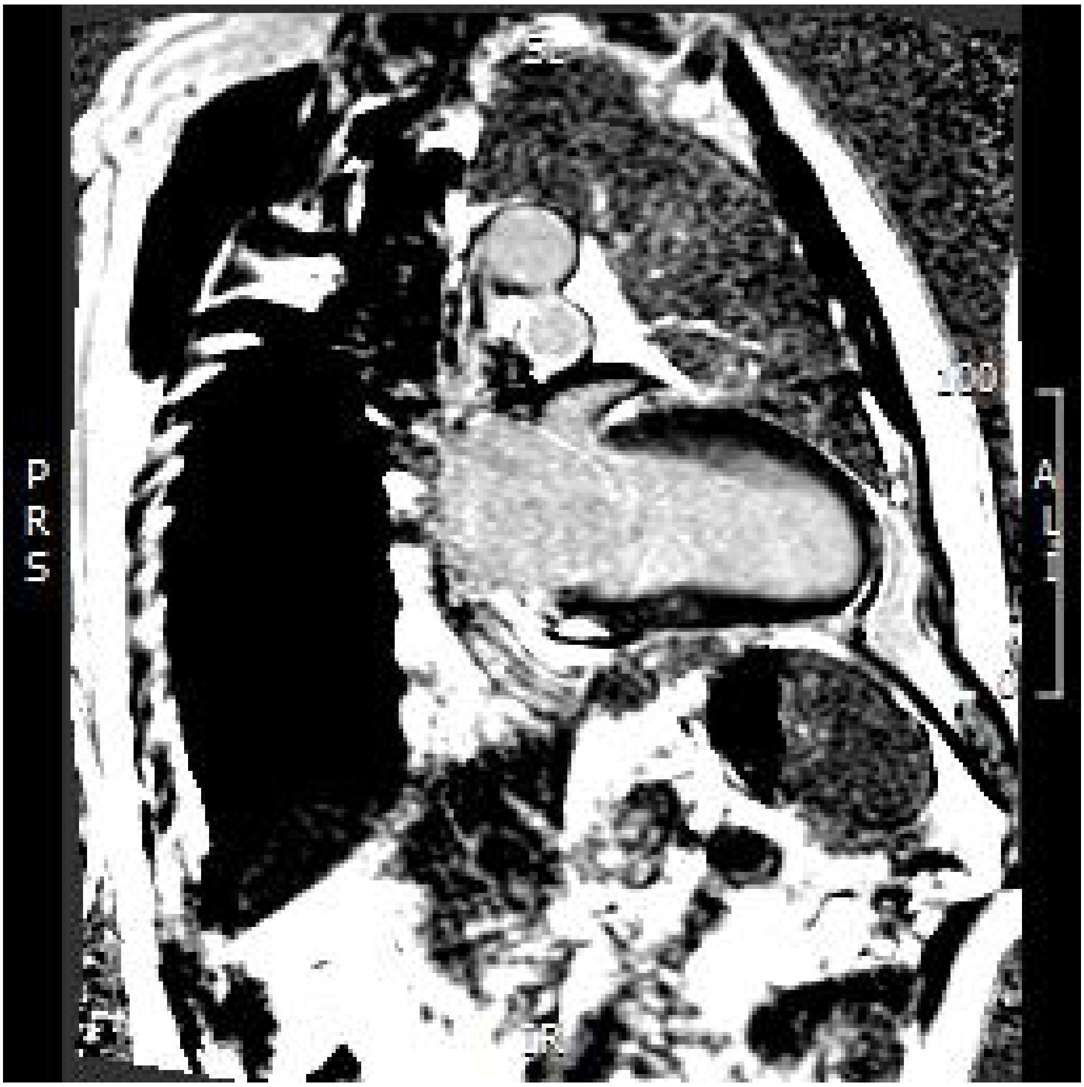
PRS
AIL
100
0

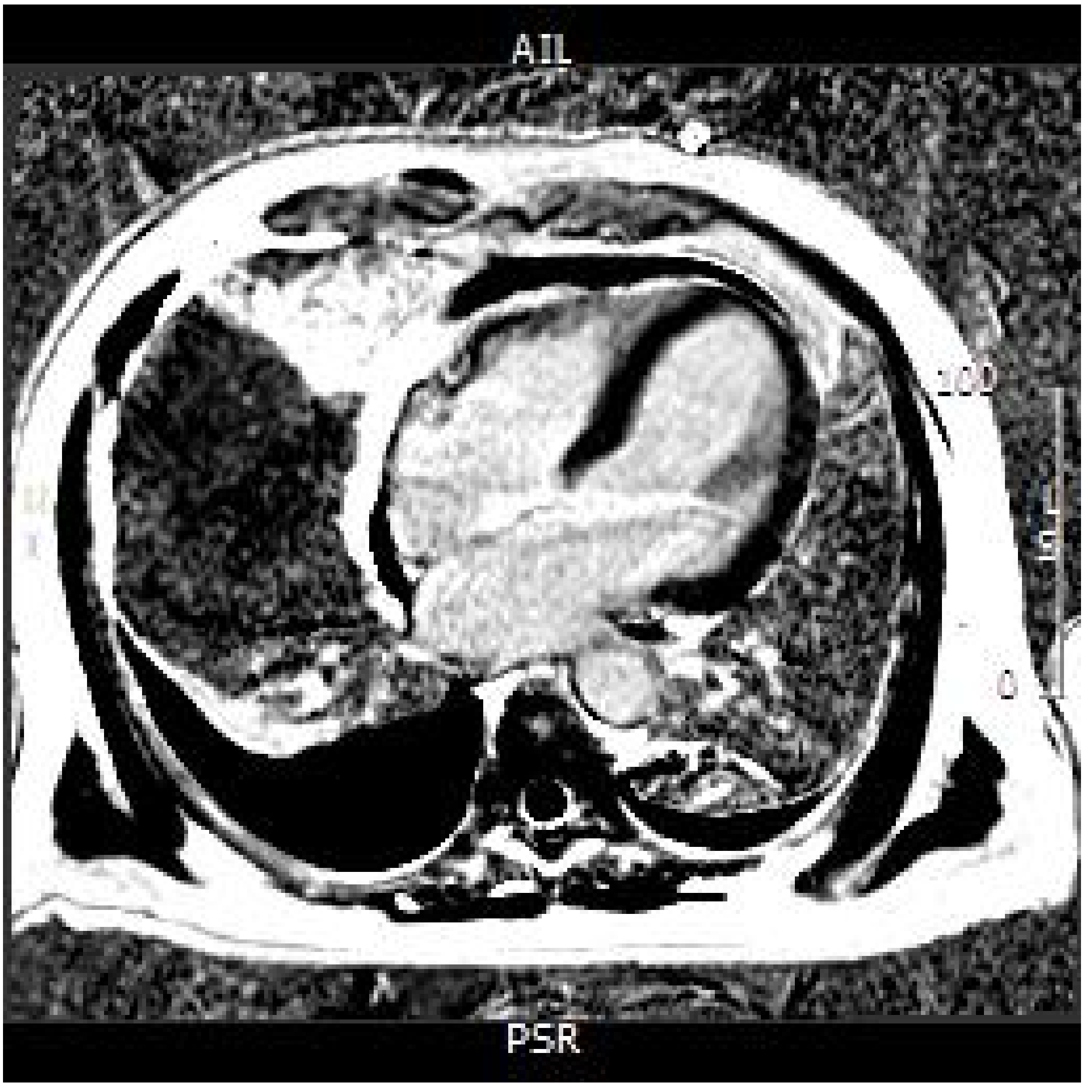
AIL
100
0
PSR

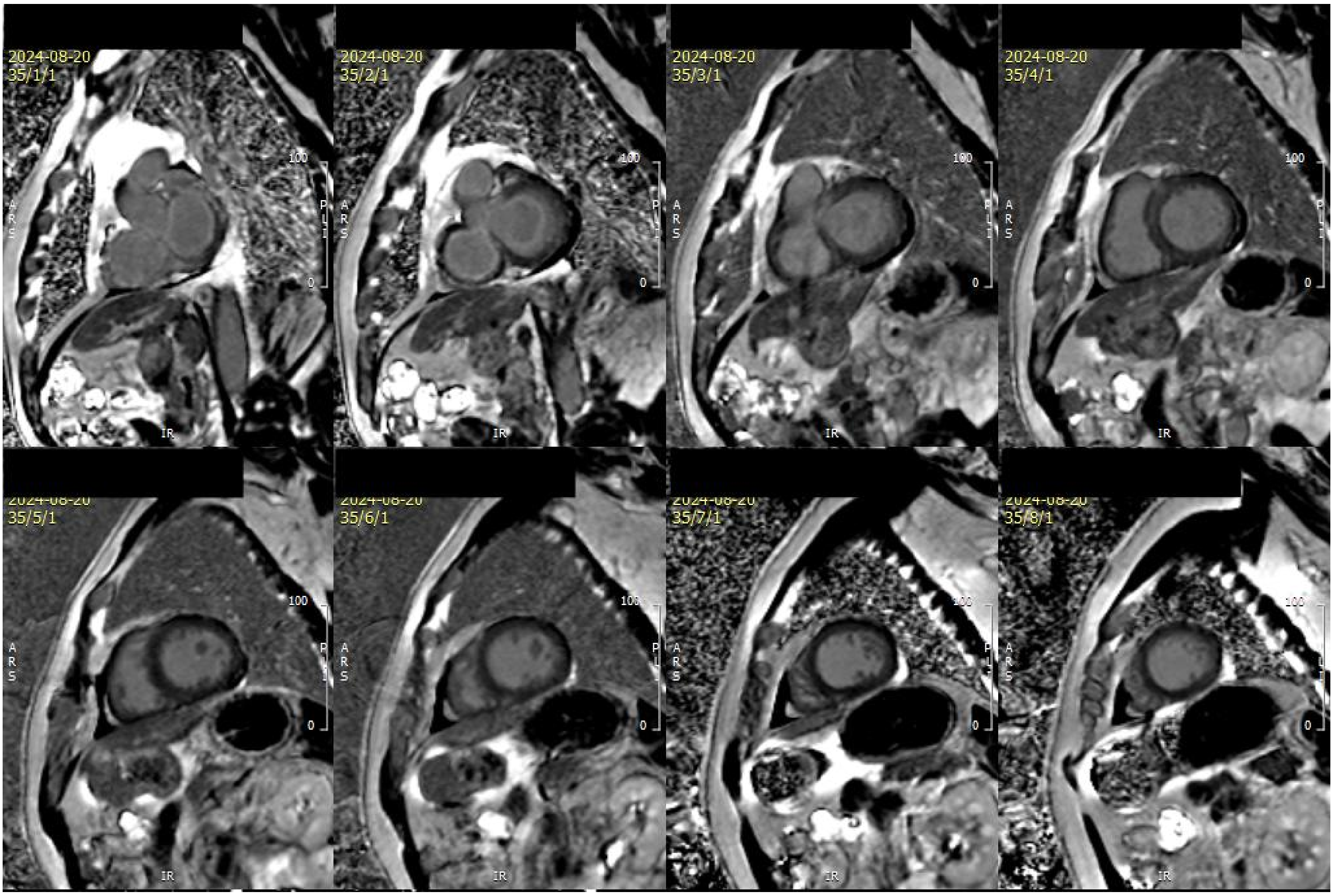

Figure S2. Cardiac magnetic resonance imaging did not reveal clear manifestations of ischemic cardiomyopathy.
